# Stronger gut microbiome modulatory effects by postbiotics than probiotics in a mouse colitis model

**DOI:** 10.1038/s41538-022-00169-9

**Published:** 2022-11-15

**Authors:** Tao Zhang, Weiqin Zhang, Cuijiao Feng, Lai-Yu Kwok, Qiuwen He, Zhihong Sun

**Affiliations:** 1 Key Laboratory of Dairy Biotechnology and Engineering, Ministry of Education, Inner Mongolia Agricultural University, 010018 Hohhot, P. R. China; 2 Key Laboratory of Dairy Products Processing, Ministry of Agriculture and Rural Affairs, Inner Mongolia Agricultural University, 010018 Hohhot, P. R. China; 3 Inner Mongolia Key Laboratory of Dairy Biotechnology and Engineering, Inner Mongolia Agricultural University, 010018 Hohhot, P. R. China

**Keywords:** Microbiology, Ulcerative colitis

## Abstract

Probiotics are increasingly used as adjunctive therapy to manage gastrointestinal diseases, such as ulcerative colitis. However, probiotic use has posed some safety concerns. Thus, postbiotics are proposed as alternatives to probiotics in clinical applications. However, no study has directly compared the clinical benefits of probiotics and postbiotics. This study compared the beneficial effect of postbiotics and probiotics derived from the strain, *Bifidobacterium adolescentis* B8589, in a dextran sulfate sodium (DSS)-induced experimental colitis mouse model. Four groups of mice (*n* = 7 per group) were included in this work: Control (received water plus saline), DSS (received DSS without postbiotic/probiotic), Postbiotic (received DSS plus postbiotic), and Probiotic (received DSS plus probiotic). Our results showed that intragastric administration of both probiotic and postbiotic ameliorated colitis, reflected by decreased histology scores in Postbiotic and Probiotic groups compared with DSS group (*P* < 0.05). The fecal microbiota alpha diversity was not significantly affected by DSS-, postbiotic, or probiotic treatment. However, the postbiotic treatment showed stronger effects on modulating the fecal microbiota beta diversity, composition, and metagenomic potential than the probiotic treatment. Overall, our findings suggested that probiotics and postbiotics had similar ability to improve disease phenotype but had distinct ability to regulate the gut microbiota and metabolic pathways in the context of ulcerative colitis. In view of the smaller safety concern of postbiotics compared with probiotics and its stronger modulatory effect on the host gut microbiota, we propose that postbiotics are to be considered for use as next-generation biotherapeutics in managing ulcerative colitis or even other diseases.

## Introduction

Probiotics are “live microorganisms which when administered in adequate amounts confer a health benefit on the host”^1^. Moreover, probiotics have been increasingly used in clinical settings in adjunct to conventional drugs for disease management^2–7^. However, there are potential safety concerns of probiotic use in patients with severe disease^8^. For example, a multicenter, randomized, double-blind, placebo-controlled trial assessed the effects of probiotics in 298 patients with severe acute pancreatitis found that the mortality of patients in the probiotic group (16%) was higher than that in the placebo group (6%)^9^. The risk of bloodstream infections occurred in six of the 522 patients (1.1%) given *Lactobacillus rhamnosus* GG compared with only two of the 21,652 patients (0.009%) who did not receive this probiotic^10^. Thus, although probiotics are generally regarded as safe, their potential health risk should be taken into account when prescribed to patients with critical illness; and this low level of risk has hindered their clinical applications as promising live biotherapeutics in specific groups of patients. In view of such limitation, it would be of interest to develop products that not only resemble the functional repertoire of probiotics but also pose no or near absence of risk to users.

In 2021, the International Scientific Association for Probiotics and Prebiotics defined postbiotics as “preparation of inanimate microorganisms and/or their components that confers a health benefit on the host”^11^. Recent evidence is beginning to show that the biological activities of postbiotics on host health may be comparable to those offered by probiotics, particularly in managing gastrointestinal disorders, even though they are devoid of live microorganisms^12^. The concern of probiotic viability is no longer an issue in the case of postbiotics, which has no risk in causing life-threatening conditions like bacteremia as in probiotic therapy^13^. Another advantage of postbiotics over probiotics is the avoidance of transmitting potential virulence genes or spreading antibiotic resistance genes from live probiotics to the gut microbiota via horizontal transfer after gaining access to the gastrointestinal tract^14^. Additionally, the postbiotic preparations can be easily and stably stored at room temperature over years without the need to consider the progressive reduction in biological activity due to loss of bacterial viability over time^11^. These functional and physical attributes of postbiotics have spurred considerable interest among investigators^15^.

Ulcerative colitis (UC) is a subtype of inflammatory bowel disease, which is a chronic inflammatory disease involving the colon and rectum that mostly manifests as abdominal pain, diarrhea, and rectal bleeding^16^. Although the etiology remains poorly understood, it is widely accepted that environmental, genetic, microbial and immune factors together contribute to the development and progression of UC^17^. Among the various factors, the role of gut microbiota has received much attention^18^. Moreover, considerable evidence indicates that probiotics administration could attenuate UC-associated symptoms and inflammation, presumably through modulating the gut microbiota (Table 1)^19–32^. Meanwhile, emerging studies also reported that postbiotic application could reduce the risk of development of UC^31,33–36^. No study has directly compared the beneficial effects of probiotics and postbiotics in parallel, which may help provide practical guiding information for designing postbiotic-based therapeutic strategies and treatment regimens.

**Table 1:** Summary of major rodent studies investigating the protective effect of probiotics against dextran sulfate sodium (DSS)-induced ulcerative colitis.

Probiotic strain	Probiotic dose	Probiotic treatment duration	Time of probiotic application	Main observations	Reference
*Akkermansia muciniphila* Muc^T^	3 × 10^9^ CFU/d	14 d	7 d before induction of colitis	Reduced weight loss, colon length shortening and histopathology scores; enhanced gut barrier function Reduced serum and tissue levels of inflammatory cytokines and chemokines Alleviated gut dysbiosis and reshaped the gut microbiota community	Bain et al.^19^
*Lactobacillus paracasei* subsp. *paracasei* NTU 101	2.3 × 10^9^ or 4.5 × 10^9^ CFU/kg BW/d	25 d	14 d before induction of colitis	Improved antioxidant capacity, reduced pro-inflammatory cytokine levels, increased anti-inflammatory cytokine levels, and slightly ameliorated body weight loss	Chen et al.^20^
*Lactobacillus plantarum* AR326	2 × 10^9^ CFU/d	7 d	6 d after induction of colitis	Reduced body weight loss, disease activity index (DAI), colon length shortening, myeloperoxidase activity and histological damage Restored the tight junction protein expression and reduced the abnormal expression of pro-inflammatory cytokines	Wang et al.^21^
*Bifidobacterium bifidum* ATCC 29521	2 × 10^8^ CFU/d	27 d	21 d before induction of colitis	Regulated the expression of immune markers and tight junction proteins in the colon Ameliorated expression of selected miRNA, including miR-150, miR-155, and miR-223 Restored healthier gut microbiota from a gut dysbiosis	Din et al.^22^
*Lactobacillus plantarum* L15	1 × 10^9^ or 1 × 10^10^ CFU/mL (1 mL/100 g BW)	28 d	7 d after induction of colitis	Increased the body weight, colon length and anti-inflammatory cytokine production Decreased pro-inflammatory cytokine production, DAI levels, and myeloperoxidase parameters Alleviated colonic histopathological changes, modulated the gut microbiota, and decreased lipopolysaccharide secretion Suppressed Toll-like receptor 4-nuclear factor-κB (TLR4-NF-κB) signaling pathway activation	Yu et al.^23^
*Lactobacillus fermentum* ZS40	1 × 10^9^ CFU/kg BW/d	35 d	21 d before induction of colitis	Reduced histopathology scores, myeloperoxidase and malondialdehyde levels Increased total superoxide dismutase and catalase in mouse serum Regulated the balance of pro-inflammatory cytokines and anti-inflammatory cytokines Inhibited the activation of nuclear factor-κB (NF-κB) and mitogen-activated protein kinase (MAPK) signaling pathways	Chen et al.^24^
*Lactobacillus casei* ATCC 393	2 × 10^9^ CFU/d	14 d	14 d before induction of colitis	Reduced body weight loss, DAI, colon length shortening, and villus height of colon tissue Inhibited the infiltration of immune cells into the intestinal mucosa, decreased the production of pro-inflammatory factors, and increased serum and colon tissue expression of anti-inflammatory factors Increased the expression levels of occludin, ZO-1, and claudin-1, while reduced the expression of nucleotide binding oligomeric domain-like receptor protein 3 (NLRP3), cysteine proteinase-1 (caspase-1), interleukin (IL)−1β, and IL-18 Improved DSS-induced gut microbiota dysbiosis	Dou et al.^25^
*Saccharomyces boulardii*	1 × 10^5^ or 1 × 10^7^ CFU/d	21 d	21 d before induction of colitis	Reduced DAI, colon length shortening, and loss of histological structure Protected the intestinal barrier, suppressed colonic inflammation, restored myeloperoxidase activity, mitigated colonic oxidative damage Suppressed the nuclear translocation of NF-κB p65 subunit, promoted the nuclear translocation of nuclear factor erythroid 2-related factor 2 (Nrf2)	Gao et al.^26^
*Bifidobacterium* *Infantis CGMCC0460.1*	1.5 × 10^9^ CFU/d	14 d	7 d before induction of colitis	Promoted the recovery of intestinal injury and modulated the gut microbiota composition Maintained genome stability partially by upregulating the expression of anaphase-promoting complex subunit 7 (APC7)	Han et al.^27^
*Akkermansia muciniphila* ATCC BAA-835	1 × 10^9^ CFU/d	7 d	7 d before induction of colitis	Decreased body weight loss, colon length shortening, and colon histological inflammatory score Enhanced the number of goblet cells and the mucin family Downregulated pro-inflammatory cytokines such as tumor necrosis factor alpha (TNF-α), IL-6, and monocyte chemoattractant protein 1 (MCP−1)	Qu et al.^28^
*Lactiplantibacillus plantarum* DMDL 9010	1 × 10^7^ or 1 × 10^9^ CFU/mL (0.2 mL/10 g BW)	7 d	At the same time	Reduced the inflammatory response, repaired intestinal barrier damage, and lightened depression-like behavior Inhibited neuroinflammation by upregulating the levels of neurotransmitters, especially 5-hydroxytryptamine, norepinephrine, dopamine, and 5-hydroxyindole-3-acetic acid Reorganized the gut microbiome and increased the levels of short-chain fatty acids (SCFAs)	Huang et al.^29^
*Lactobacillus acidophilus* ATCC 4356	1 × 10^8^ CFU/d	8 d	At the same time	Decreased DAI scores, improved colon shortening, and protected against splenomegaly and thymic atrophy Increased the contents of SCFAs, inhibited NLRP3 inflammasome and facilitated autophagy	Li et al.^30^
*Clostridium butyricum* MIYAIRI II 588	1 × 10^8^ CFU/d	21 d	21 d before induction of colitis	Prevented body weight loss, reduced DAI/colon histology scores and colon length shortening, and improved gut barrier function Reduced pathogenic bacteria and increased beneficial bacteria	Ma et al.^31^
*Companilactobacillus crustorum* MN047	1 × 10^9^ CFU/d	24 d	14 d before induction of colitis	Attenuated the increased DAI, shortened colon length, gut barrier damage, and inflammation Upregulated the expressions of MUCs and tight junctions, downregulated the expressions of pro-inflammatory cytokines and chemokines, increased fecal SCFAs, and lowered serum lipopolysaccharides Regulated gut microbiota (e.g., increased *Akkermansia*, *Blautia*, and *Ruminococcus* levels)	Wang et al.^32^

*Bifidobacterium adolescentis* B8589 is a probiotic bacterium that was isolated from an infant stool sample collected in the Inner Mongolia Autonomous Region. The strain was identified and characterized in our laboratory, and it was found to be acid and bile resistant. The metabolites of this strain have been shown to possess anti-inflammatory, antioxidant, and antibacterial properties, revealed by metabolomic studies. However, the functional effect of *Bifidobacterium adolescentis* B8589 has not previously been investigated as probiotics or postbiotics in preclinical studies of UC.

The objective of this study was to directly compare the beneficial effects of probiotics and postbiotics in mitigating dextran sulfate sodium (DSS)-induced colitis in a mouse model. The DSS-induced colitis model simulates the condition of UC, and our results confirmed that both probiotics and postbiotics could alleviate colitis-associated symptoms, reflected by the lowered colon histopathological scores in Probiotic and Postbiotic groups compared with DSS group. Postbiotics exhibited a stronger ability to modulate the gut microbiota and its functional metagenomic potential compared with probiotics. Our findings support that postbiotics are promising and safe adjuvant therapeutics alternative to probiotics for clinical use.

## Results

### Both postbiotics and probiotics administration attenuated the symptoms of DSS-induced colitis

**Fig. 1:**
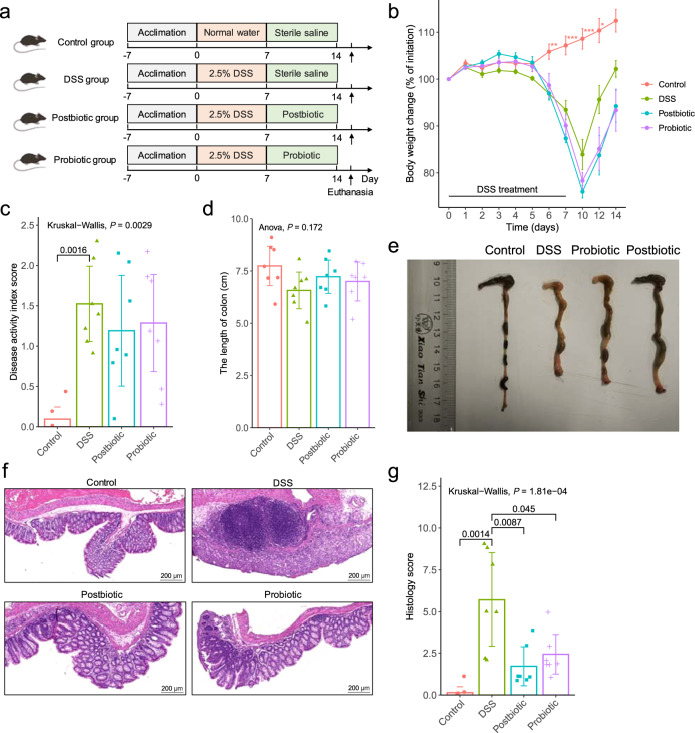
Both postbiotics and probiotics administration attenuated DSS-induced experimental colitis. **a** Schematic illustration of the experimental design. The animal trial was performed with male specific pathogen-free C57BL/6 J mice (*n* = 7 per group). All mice were acclimatized for a week prior to ulcerative colitis induction by providing dextran sulfate sodium (DSS) in drinking water. After acclimatization, the groups were randomized into four groups: Control, DSS, Postbiotic, and Probiotic, for respective interventions. **b** Body weight change of mice in the animal trial. **c** Disease activity index (DAI) scores at the end of the animal trial. **d** Colon length at the end of the animal trial. **e** Representative pictures of colon morphology of mice from the four treatment groups. **f** Representative micrographs of colon tissue sections of four groups of mice. **g** Histological scores of colon at the end of the animal trial. The error bars on the line and bar charts represented the standard deviations. The scale bar of representative images is ×40 by 200 μm. *P < 0.05, **P < 0.01, ***P < 0.001.

To compare the beneficial effect of postbiotics and probiotics application, a DSS-induced colitis model was constructed (Fig. 1a). The body weight of DSS-treated mice (including those received postbiotic or probiotic treatment) decreased apparently, especially after day 5, contrasting to significant increases in the healthy control mice between days 6 and 12 compared with baseline (*P* < 0.05), suggesting that postbiotic or probiotic administration did not significantly improved DSS-induced body weight loss (Fig. 1b). The intake of postbiotic or probiotic reduced the disease activity index (DAI) scores (Fig. 1c) and colon shortening effect (Fig. 1d, e) resulted from DSS-induction, although the differences were non-significant. However, mice received postbiotic or probiotic intervention exhibited a marked decrease in inflammatory cell infiltration, mucosal damage, and loss of crypts (Fig. 1f), which was supported by significant decreases in the histological scores (*P* < 0.05, DSS group vs Postbiotic group or Probiotic group; Fig. 1g). Collectively, these results suggested that both postbiotics and probiotics administration could alleviate some of the symptoms and pathophysiology associated with DSS-induced colitis.

### Gut microbiota diversity was regulated by postbiotic but not probiotic

**Fig. 2:**
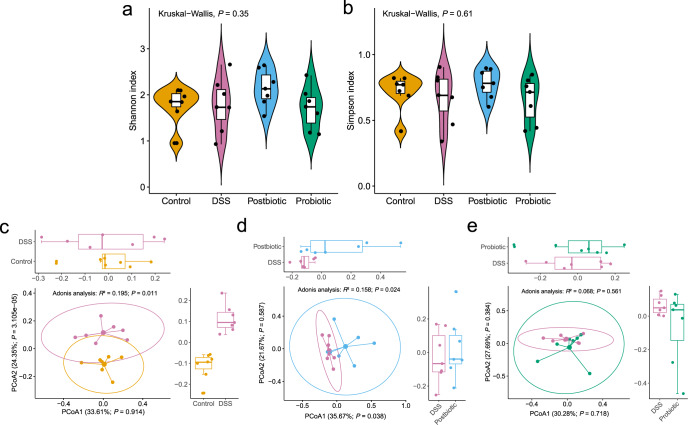
Postbiotic but not probiotic regulated the gut microbiota diversity. Shannon index (**a**) and Simpson index (**b**) of the fecal microbiota of the Control, Dextran sulfate sodium (DSS), Postbiotic, and Probiotic groups. Principal coordinate analysis (Bray–Curtis dissimilarity) score plot of species-level fecal microbiota of the Control and DSS groups (**c**), Postbiotic and DSS groups (**d**), and Probiotic and DSS groups (**e**). In the boxplot, horizontal line represents the median of the data, lower and upper bounds of the box represent the 25th and 75th percentile of data, and the whiskers represent the minimum and maximum of the data. The scattered point on the box represents the actual data points.

To analyze the effect of postbiotic or probiotic intervention on the fecal microbiota, whole-metagenome shotgun sequencing was performed on 28 fecal samples (*n* = 7 per group) collected at the end of the trial. The fecal microbiota of DSS group had numerically lower values in both Shannon and Simpson indexes, which were restored by postbiotic but not probiotic treatment (Fig. 2a, b). We then analyzed beta diversity by Principal coordinates analysis (PCoA) (Bray–Curtis dissimilarity distance), and samples representing healthy control and DSS-treated mice showed distinct clustering pattern on the PCoA score plot (Adonis test, *R*^2^ = 0.195, *P* = 0.011; Fig. 2c), suggesting apparent differences in the fecal microbiota structure between Control and DSS groups. Similar analyses were performed between DSS and Postbiotic groups, as well as DSS and Probiotic groups. Interestingly, significant difference in the fecal microbiota structure was only observed between Postbiotic and DSS groups (*R*^2^ = 0.158, *P* < 0.024; Fig. 2d) but not between that of Probiotic and DSS groups (*R*^2^ = 0.068, *P* = 0.561; Fig. 2e), suggesting that the postbiotic intervention had advantages over probiotic in restoring the gut microbiota diversity, which was disrupted by DSS treatment.

### Postbiotics had stronger ability to regulate the gut microbiota composition in DSS-induced colitis mice than probiotics

**Fig. 3:**
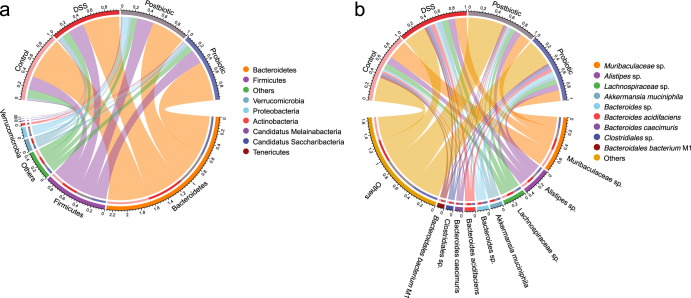
Compositional differences in fecal microbiota among Control, DSS, Postbiotic, and Probiotic groups. Chord diagram showing phylum-level (**a**) and species-level (**b**) of the fecal microbiota. The upper half circle represents the four treatment groups, while the lower half circle represents the overall composition of fecal microbiota across all groups. DSS, dextran sulfate sodium.

Given postbiotics but not probiotics had the capacity to regulate the gut microbiota diversity of mice, we then asked whether both of them could modulate the gut microbiota composition. The overall fecal metagenome dataset comprised one domain, eight phyla, 17 classes, 24 orders, 41 families, 62 genera, and 117 species. We identified eight phyla across the four groups, including Bacteroidetes, Firmicutes, Verrucomicrobia, Proteobacteria, Actinobacteria, Candidatus Melainabacteria, Candidatus Saccharibacteria, and Tenericutes. Bacteroidetes (57.24%) and Firmicutes (22.92%) were the two common top phyla across four groups (Fig. 3a). No significant difference was found in the phylum-level fecal microbiota composition between DSS and Postbiotic groups or between DSS and Probiotic groups (Supplementary Fig. 1).

**Fig. 4:**
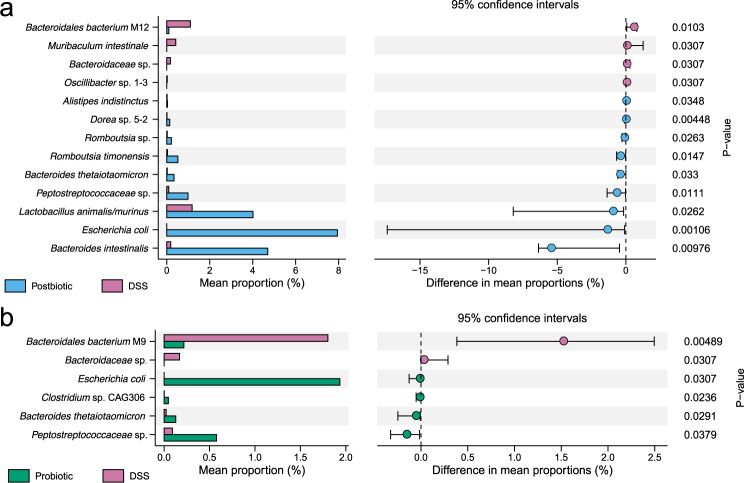
Postbiotics had a stronger effect on the gut microbiota composition in DSS-induced colitis mice compared with probiotics. The differential species between the Postbiotic group and Dextran sulfate sodium (DSS) group (**a**) and between the Probiotic group and DSS group (**b**), evaluated by Wilcoxon rank-sum test.

At the species level, a total of 117 species were identified across all samples, and the most dominant species were *Muribaculaceae* sp. (19.93%), *Alistipes* sp. (10.24%), *Lachnospiraceae* sp. (8.10%), *Akkermansia muciniphila* (4.45%), *Bacteroides* sp. (4.12%), and *Bacteroides acidifaciens* (3.88%) (Fig. 3b). Thirteen and six differential species were identified between Postbiotic and DSS groups and between Probiotic and DSS groups, respectively (Fig. 4a, b). Of note, both Postbiotic and Probiotic groups had significantly fewer *Bacteroidaceae* sp. (*P* < 0.05) but significantly more *Escherichia coli*, *Peptostreptococcaceae* sp., and *Bacteroides thetaiotaomicron* (*P* < 0.05) compared with DSS group. In addition, Postbiotic group had significantly more *Bacteroides intestinalis*, *Lactobacillus animalis/murinus*, and *Romboutsia timonensis* (*P* < 0.05), but fewer *Bacteroidales bacterium* M12, *Muribaculum intestinale*, and *Bacteroidaceae* sp. (*P* < 0.05) compared with DSS group. Collectively, these results suggested that postbiotics had a stronger effect on the gut microbiota composition in DSS-induced colitis mice compared with probiotics.

### Both postbiotic and probiotic supplementation modulated the functional potential of the gut microbiome

**Fig. 5:**
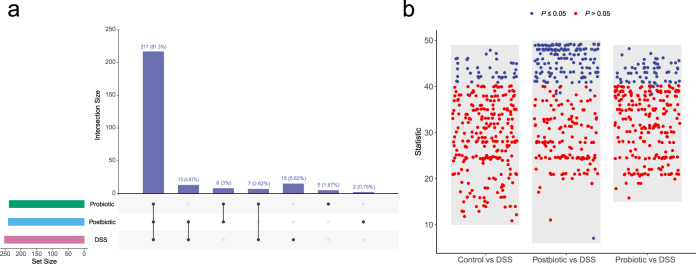
Postbiotic and probiotic supplementation modulated the functional potential of the gut microbiome. **a** Number of metabolic pathways identified in the fecal metagenome of the Postbiotic, Probiotic, and Dextran sulfate sodium (DSS) groups (left panel). The right panel shows common pathways between datasets. **b** Number of differential metabolic pathways between groups: Control, Postbiotic, Probiotic groups versus DSS group. Differential metabolic pathways were detected by Wilcoxon rank-sum test.

We then compared the gut metagenomic potential across four groups by HUMAnN2 pipeline using default settings and through the MetaCyc database^37^. Of the 286 metabolic pathways identified across all samples, 217 (81.3%) were common to DSS, probiotic, and postbiotic groups, while 15 (5.62%), 5 (1.87%), and 2 (0.75%) were unique to DSS, probiotic, and postbiotic groups, respectively (Fig. 5a). We then identified differential metabolic pathways between DSS and the other three groups by Wilcoxon rank-sum test, returning: 40, 144, and 61 between DSS and Control groups (Supplementary Table 1), DSS and Postbiotic groups (Supplementary Table 2), and DSS and Probiotic groups (Supplementary Table 3), respectively (Fig. 5b). These results demonstrated that administering postbiotic and probiotic, especially the former, could modulate the functional potential of the gut microbiome.

### Postbiotic and probiotic mediated divergent changes in associations between gut microbiota and metabolic pathways

**Fig. 6:**
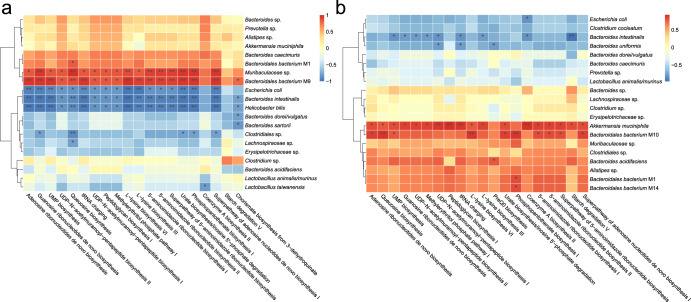
Postbiotics and probiotics caused divergent changes in the intragroup associations between fecal microbes and metabolic pathways. Spearman’s correlation heatmap of top 20 bacterial species and metabolic pathways in the fecal microbiota of Postbiotic group (**a**) and Probiotic group (**b**) at the end of the animal trial. The color intensity is proportional to the strength of Spearman’s correlation. *P < 0.05; **P < 0.01.

Correlation analysis was performed to compare postbiotic- and probiotic-induced intergroup changes in the associations between the fecal microbes and their encoded metabolic pathways. Spearman’s correlation analysis of the top 20 fecal microbial species and 20 encoded metabolic pathways of the postbiotic-fed mice (Fig. 6a) revealed significant positive correlations between most of the encoded metabolic pathways with *Bacteroidales bacterium* M9 and *Muribaculaceae* sp., while negatively correlated with *Helicobacter bilis*, *Bacteroides intestinalis*, and *Escherichia coli*.

Conversely, fewer and weaker intragroup correlations were observed between the fecal microbes and encoded metabolic pathways in the probiotic-fed mice (Fig. 6b). Generally, the encoded metabolic pathways in the probiotic-fed mice had most positive correlations with *Akkermansia muciniphila* and *Bacteroidales bacterium* M10, while having most negative correlations with *Bacteroides intestinalis* and *Bacteroides uniformis*.

The postbiotic and probiotic groups shared 16 bacterial species, seven of which exhibited incongruent correlations with most of the top 20 encoded metabolic pathways (Supplementary Table 4). Specifically, *Bacteroides acidifaciens*, *Clostridiales* sp., *Clostridium* sp., *Erysipelotrichaceae* sp., and *Lachnospiraceae* sp. showed negative correlations with the encoded pathways in the postbiotic-fed mice, but positive correlations were observed in the probiotic-fed mice. On the other hand, *Bacteroides caecimuris* and *Prevotella sp*. showed positive correlations with the top 20 encoded pathways in the postbiotic-fed mice, whereas negative correlations were observed in the probiotic-fed mice.

These data supported that the postbiotics and probiotics caused divergent changes in the intragroup associations between fecal microbes and metabolic pathways.

## Discussion

UC is an inflammatory bowel disease. Previous studies showed that probiotic application could ameliorate the symptoms and pathphysiology of UC (summarized in Table 1). Recently, the potential of using postbiotic alternatively to probiotic has drawn much attention due to safety concern of probiotic application in severely ill patients^38^. However, few works have directly compared the therapeutic effect of probiotic and postbiotic application. Thus, this study compared the beneficial effects of probiotics and postbiotics (both comprised solely of the probiotic strain *Bifidobacterium adolescentis* B8589) in a DSS-induced UC mouse model.

Here, we found that both postbiotics and probiotics administration protected against DSS-induced colitis, reflected by the decreased histology scores in Postbiotic and Probiotic groups compared with DSS group. However, neither the postbiotic nor probiotic application significantly affected weight loss, DAI, and colon length in mice with colitis, which were inconsistent with previous findings showing protective effects in these aspects^39–41^. The discrepant results between this and previous studies in the protective effect against DSS-induced colitis could be due to the different strains of postbiotics and probiotics used and different experimental design between studies. Moreover, since body weight loss is one of the criteria that determines the DAI score, it is also responsible for the non-significant difference in DAI observed in this study.

The most interesting observation of this work was that although both the postbiotic and probiotic showed similar potential in attenuating colitis symptoms, their capacity of regulation of gut microbial diversity was different. Although no significant difference was observed in the alpha diversity between the fecal microbiota of DSS group compared with Postbiotic or Probiotic group, administering postbiotic but not probiotic regulated the beta diversity of the fecal microbiota of mice with colitis. Moreover, the postbiotic application also showed stronger capacity modulating the gut microbiota composition compared with the probiotic intake.

Differences in the gut microbiota modulatory effect between *Bifidobacterium adolescentis* B8589 and probiotic strains used in previous fecal microbiome-based studies in experimental colitis mouse model are summarized in Supplementary Table 5^19,22,25,29,31,32,41–45^. Our results were similar to the observation of several previous reports that the phylum-level fecal microbiota did not show obvious changes subjected to probiotic supplementation^22,29,31,32,41,44^. Two studies identified significant changes in the species-level fecal microbiota. One study observed marked increases in levels of *Muribaculaceae* uncultured bacterium and *Odoribacter* unclassified, while significant decreases in levels of *Lachnospiraceae* NK4A136 group uncultured bacterium and *Mucispirillum* uncultured bacterium^22^. The other study reported dramatic increases in the abundance of *Akkermansia muciniphila* and *Escherichia coli* group^45^. In our dataset, *Muribaculaceae* sp. and *Akkermansia muciniphila* were dominant species across the four groups, and *Escherichia coli* was differentially enriched in Probiotic group compared with DSS group. In addition, previous studies found that *Akkermansia muciniphila* and *Escherichia coli* played a crucial role in the development and progression of inflammatory bowel disease^46,47^. Thus, the role of these microbes warrants further investigation. A paucity of studies has assessed the effect of postbiotics on the clinical outcome in UC, and continual efforts should be made to interrogate their role in UC.

Changes in the gut microbiota composition would naturally be accompanied by modulation in its encoded metabolic pathways and colonic metabolome^48,49^. Consistently, our results showed that postbiotic and, to a lesser extent, probiotic applications mediated changes in the gut metagenomic potential. More differential metabolic pathways were identified in Postbiotic group compared with Probiotic group.

Overall, our direct comparison of postbiotic and probiotic revealed that both of them conferred a certain extent of UC-associated symptom alleviation effects, but the postbiotic exerted stronger capacities in mitigating UC and modulating the gut microbiome than probiotic. The only difference between probiotics and postbiotics was the viability of the bacterial cells. As mentioned earlier, the use of postbiotics offer several advantages over probiotics, e.g., potential safety concerns of spreading of the intrinsic antibiotic resistance and other virulence genes in live probiotics to the gut microbes or the very slim chances of gaining access to extraintestinal organs and causing life-threatening conditions like septicemia. Thus, in addition to these advantages, the stronger protective effect of postbiotics against UC-associated symptoms observed in this study supported that they could be promising alternatives to probiotics to be applied in clinical settings and critically ill patients.

To the best of our knowledge, this is the first study to systematically compare the performance of probiotics and postbiotics derived from the same strain in an experimental colitis model. Nevertheless, we acknowledge some limitations. First, despite the fact that probiotics and postbiotics affected the gut microbiota, the exact mechanisms by which probiotics and postbiotics modulated the health status of the diseased mice remain obscure. Second, while postbiotics are generally thought to have less safety risks than probiotics^12^, the current study did not provide strong experimental evidence to support this hypothesis. Large-scale preclinical animal models and high-quality human clinical studies are still needed to disentangle the underlying mechanisms and validate this hypothesis.

In parallel, our research raises several knowledge gaps in postbiotic and/or probiotic application in managing diseases UC. First, the dose, duration, and timing of probiotics and/or postbiotics, as well as the dose of drug-induced DSS model differ among laboratories, and such difference might have caused discrepant results and obscured the bona fide efficacy of probiotics and/or postbiotics. An elegant approach to tackle this conundrum is to establish a universal assessment standard for probiotics and/or postbiotics. Additionally, uniform criteria for animal model construction are also required. Second, the strain specificity of probiotics and/or postbiotics in managing colitis or other diseases varies^42,50^. Given the strain specificity, continual efforts should be made to isolate, screen, and characterize high-potency probiotics and/or postbiotics for clinical use. Third, one of the most commonly used methods for microbial enumeration is plate count, which expresses the results in the form of live microorganisms in colony-forming units. Indeed, dead cells are inevitable in live probiotic preparation, and it is likely that the beneficial effects could be resulted from both viable and dead cells in the preparation. Thus, for precise analysis and clear distinction of the functional differences between postbiotics and probiotics, it would be necessary to accurately enumerate the relative proportions of live and dead cells in a probiotic culture^51^. Fourth, given that both dead and live microorganisms exerted similar beneficial effects, the key scientific question remains to be answered is whether the beneficial effects exerted by probiotics are in fact contributed solely by the living microorganisms or also by the yet to be clarified mechanism of action by the dead cells in the same preparation.

In conclusion, our findings highlight the differential function of probiotics and postbiotics in colitis remission, providing insights into the role of probiotic and postbiotic products in health improvement; and it would be of interest to further explore the exact mechanisms of action of probiotics and postbiotics. The findings of our study support that postbiotics could be prioritized over probiotics for use in disease management due to their minimal safety concerns.

## Methods

### Animals

Male specific pathogen-free (SPF) C57BL/6 J mice (age 6–8 weeks, weight 18–22 g) were purchased from Beijing Huafukang Biotechnology Co., Ltd. (SCXK 2019-0008). All mice were housed in a standard SPF environment in the animal house of the Key Laboratory of Dairy Biotechnology and Engineering, Ministry of Education, Inner Mongolia Agricultural University (granted the laboratory animal use permit, SYXK 2020-0002). Three to four mice were maintained in each individually ventilated cage (temperature, 22 ± 2 °C; relatively humidity, 45 ± 10%; standard 12 h/12 h light/dark cycle). All mice were acclimatized one week before the formal experiment with free access to food and water ad libitum. All animal experimental protocols were strictly performed in accordance with the provisions of the National Institutes of Health of the United States, approved by the Experimental Animal Ethics Committee of the Inner Mongolia Agricultural University.

### Probiotics and postbiotics

Packaged live *Bifidobacterium adolescentis* B8589 powder (probiotics) and non-viable *Bifidobacterium adolescentis* B8589 powder (postbiotics) were provided by the Key Laboratory of Dairy Biotechnology and Engineering, Ministry of Education, Inner Mongolia Agricultural University, China.

### Treatment groups

The mouse UC model was induced by administering DSS (2.5%, w/v; molecular mass 36–40 kDa; MP Biologicals, Solon, USA) in their drinking water ad libitum for seven days. After 1 week of acclimation, mice were randomly divided into four treatment groups, as follows: (1) Control group: tap water supplemented without 2.5% DSS for seven days, followed by daily oral administration of 0.2 mL sterile saline for seven days; (2) DSS group: tap water supplemented with 2.5% DSS for 7 days, followed by daily oral administration of 0.2 mL sterile saline for seven days; (3) Postbiotic group: tap water supplemented with 2.5% DSS for 7 days, followed by daily oral administration of 0.2 mL postbiotics-containing sterile saline (2 × 10^9^ cell/d) for seven days; (4) Probiotic group: tap water supplemented with 2.5% DSS for 7 days, followed by daily oral administration of 0.2 mL probiotics-containing sterile saline (2 × 10^9^ CFU/d) for seven days.

Body weight was measured daily throughout the animal trial. The parameters of weight loss, stool consistency, and fecal occult blood were used to calculate the DAI score (Supplementary Table 6)^52^. Mice were sacrificed after anesthesia on day 15, and the length of the colon was measured. A segment of the mid-colon was flushed with sterile water and then fixed in 4% paraformaldehyde for subsequent histopathological analysis. The fecal samples of all mice were collected aseptically and stored at −80 °C for subsequent metagenomics analyses.

### Histopathological analysis

For histological assessment, paraformaldehyde-fixed colon tissues embedded in paraffin and sectioned into 4 µm-thick sections. Paraffin sections were dewaxed and stained with hematoxylin and eosin. The histological score was determined according to criteria in Supplementary Table 7^53^.

### Fecal genomic DNA extraction and whole-metagenome shotgun sequencing

The metagenomic DNA from each fecal sample was extracted using a QIAamp Fast DNA Fecal Mini Kit (Qiagen GmbH, Hilden, Germany) according to the manufacturer’s instructions. Whole-metagenome shotgun sequencing was performed using an Illumina NovaSeq 6000 platform (Illumina, San Diego, USA). Libraries were constructed using the NEBNext Ultra DNA Library Prep Kit for Illumina (NEB, Ipswich, USA), following the manufacturer’s recommendations to generate DNA fragments of ~300 bp. Paired-end reads were generated by sequencing 150 bp in both forward and reverse directions. Raw reads were quality-controlled using KneadData (http://huttenhower.sph.harvard.edu/kneaddata) and were subsequently aligned to the mouse genome to remove the host DNA fragments using Bowtie2 under default parameters^54^. Metagenomic species were annotated by mOTUs2^55^, while the functional metagenome and corresponding metabolic pathways were annotated by HUMAnN2^56^ with the UniRef90 database^57^.

### Statistical analysis

All statistical analysis and graphical visualization were performed using R software. PCoA was performed and visualized using the R package vegan and ggpubr, and the *P*-value was determined using Adonis test based on 999 permutations. Student’s *t*-test or Wilcoxon rank-sum test was used to compare differences between groups. Data from more than two groups were compared using one-way analysis of variance followed by Student’s *t*-test or Kruskal–Wallis test followed by Wilcoxon rank-sum test. Data were expressed as mean ± standard deviation. *P* < 0.05 was considered statistically significant.

### Reporting summary

Further information on research design is available in the Nature Research Reporting Summary linked to this article.

## Supplementary information

Supplementary Material

Supplementary Table 1

Supplementary Table 2

Supplementary Table 3

Reporting Summary

## Data Availability

The raw sequences reported in this article were deposited in the NCBI Sequence Read Archive under the accession number PRJNA863452.
