# Author Correction: Stronger gut microbiome modulatory effects by postbiotics than probiotics in a mouse colitis model

**DOI:** 10.1038/s41538-023-00179-1

**Published:** 2023-02-03

**Authors:** Tao Zhang, Weiqin Zhang, Cuijiao Feng, Lai-Yu Kwok, Qiuwen He, Zhihong Sun

**Affiliations:** 1 Key Laboratory of Dairy Biotechnology and Engineering, Ministry of Education, Inner Mongolia Agricultural University, 010018 Hohhot, P. R. China; 2 Key Laboratory of Dairy Products Processing, Ministry of Agriculture and Rural Affairs, Inner Mongolia Agricultural University, 010018 Hohhot, P. R. China; 3 Inner Mongolia Key Laboratory of Dairy Biotechnology and Engineering, Inner Mongolia Agricultural University, 010018 Hohhot, P. R. China

**Keywords:** Microbiology, Ulcerative colitis

Correction to: *npj Science of Food* 10.1038/s41538-022-00169-9, published online 15 November 2022

The name of the strain Bifidobacterium adolescentis B8588 should be changed to Bifidobacterium adolescentis B8589 in the online version of the article. The original article has been corrected.

