# Supplementary material for: Stronger gut microbiome modulatory effects by postbiotics than probiotics in a mouse colitis model
Source: NPJ Sci Food. 2022 Nov 15;6:53. doi: 10.1038/s41538-022-00169-9 (PMC9666507; doi:10.1038/s41538-022-00169-9)
Supplement: Supplementary file 1 — Supplementary Material [file 41538_2022_169_MOESM1_ESM.pdf]

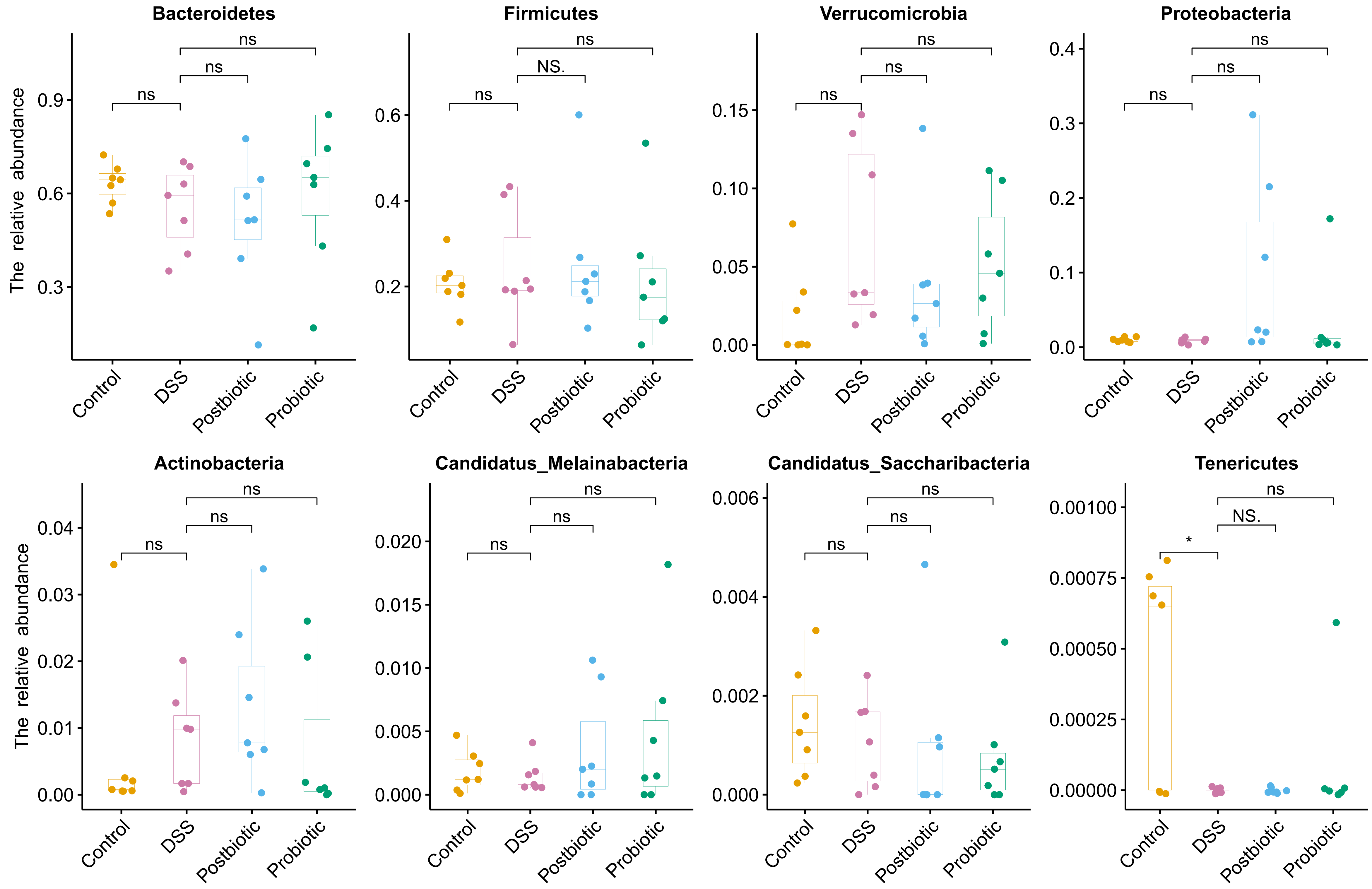

Supplementary Figure 1. Phylum-level comparison of the fecal microbiota. Differences in the relative abundance were evaluated between the dextran sulfate sodium (DSS) group and the control/postbiotic/probiotic groups were evaluated with Wilcoxon rank-sum tests, respectively (* $P < 0.05$). In the boxplot, horizontal line represents the median of the data, lower and upper bounds of the box represent the 25th and 75th percentile of data, and the whiskers represent the minimum and maximum of the data. The scattered point on the box represents the actual data points.

Supplementary Table 4. Correlation between 16 common bacterial species and their encoded top 20 metabolic pathways

Number	Common bacterial species	Postbiotic group	Probiotic group
1	*Akkermansia muciniphila*	+	+
2	*Alistipes* sp.	+	+
3	*Bacteroidales bacterium* M1	+	+
4	*Bacteroides* sp.	+	+
5	*Muribaculaceae* sp.	+	+
6	*Bacteroides dorei/vulgatus*	–	–
7	*Bacteroides intestinalis*	–	–
8	*Escherichia coli*	–	–
9	*Lactobacillus animalis/murinus*	–	–
10	*Bacteroides acidifaciens*	–	+
11	*Bacteroides caecimuris*	+	–
12	*Clostridiales* sp.	–	+
13	*Clostridium* sp.	–	+
14	*Erysipelotrichaceae* sp.	–	+
15	*Lachnospiraceae* sp.	–	+
16	*Prevotella* sp.	+	–

Note: +, positive association; –, inverse association.

Supplementary Table 5. Summary of findings from next generation sequencing-based fecal microbiome studies involving probiotic intervention in experimental colitis mouse model

**Study**	Bain et al. (2019)[19]	Din et al. (2020)[22]	Sun et al. (2020)[42]	Liu et al. (2020)[43]	Dou et al. (2021)[25]	Sun et al. (2021)[41]	Hu et al. (2022)[44]	Huang et al. (2022)[29]	Lee et al. (2022)[45]	Ma et al. (2022)[31]	Wang et al. (2022)[32]
**Alpha diversity (Measure of microbiome diversity/richness between probiotic and DSS groups) [SD = significant difference; ND = no significant difference; NR = not reported]**											
**Alpha diversity**	SD	ND	SD	ND	SD	SD	ND	SD	ND	ND	SD
**Beta diversity (Dissimilarity in overall microbiome composition between probiotic and DSS groups) [SD = significant difference; ND = no significant difference]**											
**Beta diversity**	SD	NR	SD	SD	SD	ND	ND	SD	SD	SD	SD
**Differentially abundant microbial features at phylum level [↑ = increased in probiotic group; ↓ = decreased in probiotic group]**											
**Phylum level**		ND		NR	NR	ND	ND	ND		ND	ND
Actinobacteria											
Bacteroidetes	↓										
Campilobacterota											
Cyanobacteria											
Deferribacteres											
Desulfobacterota											
Epsilonbacteraeota											
Firmicutes	↑		↑								
Fusobacteria											
Patescibacteria											
Planctomycetes											
Proteobacteria			↓						↑		
Saccharibacteria											
Tenericutes											

Verrucomicrobia	↑								↑		
**Differentially abundant microbial features at species level [↑ = increased in probiotic group; ↓ = decreased in probiotic group]**											
**Species level**	NR		NR	NR	NR	NR	NR	NR		NR	NR
Lachnospiraceae NK4A136 group uncultured bacterium		↓									
Mucispirillum uncultured bacterium		↓									
Odoribacter unclassified		↑									
Muribaculaceae uncultured bacterium		↑									
Clostridium cocleatum											
Akkermansia muciniphila									↑		
Escherichia coli group									↑		

Supplementary Table 6. Criteria for scoring disease activity index

Score	Weight loss (%)	Stool consistency	Occult blood or gross bleeding
0	None	Normal	Negative
1	1–5	Loose stool	Negative
2	5–10	Loose stool	Hemoccult positive
3	10–15	Diarrhea	Hemoccult positive
4	>15	Diarrhea	Gross bleeding

Note: disease activity index = (combined score of weight loss, stool consistency, and occult blood or gross bleeding)/3

Supplementary Table 7. Histological grading of colitis

Score	Severity of inflammation	Depth of injury	Crypt damage
0	None	None	None
1	Slight	Mucosa	Basal 1/3 damaged
2	Moderate	Mucosa and submucosa	Basal 2/3 damaged
3	Severe	Transmural	Only surface epithelium intact
4	–	–	Entire crypt and epithelium lost

Note: the histological score for each mouse was a sum of the score of severity of inflammation, depth of injury, and crypt damage.
